# Therapeutic potential of ixmyelocel-T, an expanded autologous multicellular therapy for treatment of ischemic cardiovascular diseases

**DOI:** 10.1186/s13287-015-0007-3

**Published:** 2015-03-13

**Authors:** Kelly J Ledford, Nikki Murphy, Frank Zeigler, Ronnda L Bartel, Ross Tubo

**Affiliations:** Aastrom Biosciences, Domino’s Farms, Lobby K 24 Frank Lloyd Wright Drive, Ann Arbor, MI 48105 USA

## Abstract

**Introduction:**

Bone marrow derived cellular therapies are an emerging approach to promoting therapeutic angiogenesis in ischemic cardiovascular disease. However, the percentage of regenerative cells in bone marrow mononuclear cells (BMMNCs) is small, and large amounts of BMMNCs are required. Ixmyelocel-T, an expanded autologous multicellular therapy, is manufactured from a small sample of bone marrow aspirate. Ixmyelocel-T contains expanded populations of mesenchymal stromal cells (MSCs) and M2-like macrophages, as well as many of the CD45+ cells found in the bone marrow. It is hypothesized that this expanded multi-cellular therapy would induce angiogenesis and endothelial repair.

**Methods:**

A rat model of hind limb ischemia was used to determine the effects of ixmyelocel-T on blood flow recovery. To further determine the effects on endothelial cells, ixmyelocel-T was co-cultured with human umbilical vein endothelial cells (HUVEC) in non-contacting Transwell® inserts.

**Results:**

Co-culture of HUVECs with ixmyelocel-T resulted secretion of a variety of pro-angiogenic factors. HUVECs stimulated by ixmyelocel-T exhibited enhanced migration, proliferation, and branch formation. Ixmyelocel-T co-culture also resulted in increased endothelial nitric oxide synthase (eNOS) expression and nitric oxide (NO) production. In tumor necrosis factor alpha (TNFα)-stimulated HUVECs, ixmyelocel-T co-culture decreased apoptosis and reactive oxygen species generation, increased super oxide dismutase activity, and decreased nuclear factor kappa B (NFκB) activation. Treatment with ixmyelocel-T in a rat model of hind limb ischemia resulted in significantly increased blood flow perfusion and capillary density, gene expression and plasma levels of the anti-inflammatory cytokine interleukin (IL)-10, plasma nitrates, plasma platelet-derived growth factor (PDGF)-BB, vascular endothelial growth factor (VEGF) expression, and significantly decreased plasma thiobarbituric acid reactive substances (TBARS).

**Conclusions:**

This work demonstrates that ixmyelocel-T interacts with endothelial cells in a paracrine manner, resulting in angiogenesis and endothelial protection. This data suggests that ixmyelocel-T could be useful for promoting of angiogenesis and tissue repair in ischemic cardiovascular diseases. In conclusion, ixmyelocel-T therapy may provide a new aspect of therapeutic angiogenesis in this patient population where expanded populations of regenerative cells might be required.

## Introduction

Ischemic cardiovascular (CV) disease, a leading cause of mortality in the western world, is a systemic inflammatory disease involving dysfunction of the endothelium. The disease can lead to development of peripheral arterial disease, an atherosclerotic disease that results in inadequate blood flow to the extremities and eventually critical limb ischemia (CLI) [1,2]. The prevalence of peripheral arterial disease is rapidly increasing, and currently affects 8 to 12 million people in the United States [3]. Patients with CLI often have poor prognosis, leading to amputation of the lower extremities [1]. Several alternative approaches are currently under investigation for the treatment of CLI since only partial or short-term benefits are exhibited in current interventional therapies [1,2]. One alternative approach to treating CLI is the intramuscular administration of bone marrow-derived cellular-based therapies. These therapies are thought to contribute to tissue regeneration and repair in areas of tissue ischemia predominantly through secretion of paracrine factors [4,5]. Unfortunately, the percentage of regenerative cells in bone marrow mononuclear cells (BMMNCs) is small, and a large amount of BMMNCs is required in order to induce restorative angiogenesis [6]. Efforts are therefore needed to identify novel cellular therapies consisting of expanded populations of regenerative cells that have the ability to promote therapeutic angiogenesis in advanced disease states.

Ixmyelocel-T is an expanded, autologous multicellular therapy containing a mixture of cell types cultured from BMMNC [2,7]. Ixmyelocel-T is manufactured from a small sample of bone marrow aspirate expanded for 12 ± 1 days in a fully closed automated bioreactor system, and is composed of a mixture of cells – including myeloid cells (macrophages, granulocytes, monocytes, and mixed myeloid progenitors), lymphoid cells (T cells, B cells, and a mixture of lymphoid precursors) and mesenchymal stromal cells (MSCs) [7]. The process used to generate this cell therapy expands both the CD90^+^ MSCs and CD14^+^ macrophages, while retaining many of the CD45^+^ cells found in the bone marrow since the process does not utilize any purification or enrichment steps, other than phenotypic expansion [7]. The CD14^+^ macrophages are M2-like, with expression of two M2 surface receptors CD206 and CD163 [7,8]. Recent clinical trials evaluating ixmyelocel-T therapy in the treatment of the ischemic CV diseases dilated cardiomyopathy and CLI have shown clinical promise [2,9]. A phase 2a study in dilated cardiomyopathy (the IMPACT-DCM trial) demonstrated that ischemic patients receiving ixmyelocel-T experienced a lower percentage of major adverse cardiac events compared with control [7]. A phase 2b study in CLI (the RESTORE-CLI trial) demonstrated that treatment with ixmyelocel-T was safe and improved the time to first occurrence of treatment failure [2,7]. It is hypothesized that this expanded multicellular therapy has the potential to be a promising treatment in ischemic CV diseases where angiogenesis and tissue repair are essential.

In the present study, a rat model of hind-limb ischemia and human umbilical vein endothelial cells (HUVECs) were utilized to determine whether ixmyelocel-T promotes angiogenesis and regeneration. Specifically, the ability of ixmyelocel-T to restore blood flow and promote angiogenesis *in vivo* and *in vitro* was examined.

## Methods

### Cell culture

HUVECs (Lonza Inc., Walkersville, MD, USA) were cultured in EGM-2 (Lonza, Inc.). HUVECs between passages 3 and 8 were used in the following experiments. For the generation of ixmyelocel-T, a small volume (~50 ml) of commercially available whole bone marrow (Lonza Inc.) was obtained from healthy donors, under informed consent, through needle aspiration of the posterior iliac crest, and stored in heparinized tubes during shipment at ambient temperature to a central processing facility. The mononuclear cell fraction was obtained via an automated, closed-system, Ficoll-based density gradient centrifugation separation process. The isolated mononuclear cells were then transferred to a sterile, single-use cell bioreactor cassette [7,10]. This proprietary system controlled temperature, culture medium exchange, and gas exchange during the culture period. After approximately 12 days, the cells were washed and harvested from the cassette by a multistep, automated process, and ready for experimental study. All experiments were performed with multiple donors, with each donor being considered one number.

### Rat hind-limb ischemia model

All procedures were conducted in accordance with the current guidelines for animal welfare (Guide for the Care and Use of Laboratory Animals, 1966). The procedures used in this study were reviewed and approved by the Michigan State University In Vivo Pharmacology Facility’s Institutional Animal Care and Use Committee. Eight-weeks-old male RNU Nude rats (purchased from Charles River, Horsham, Pennsylvania, USA) underwent unilateral hind-limb ischemia. In brief, under anesthesia an incision was made along the inguinal groove and the femoral artery was isolated from its origin at the external iliac artery to the distal segment where it bifurcates in the popliteal and saphenous arteries. The artery was ligated proximally and distally with a silk suture and a portion of the artery (<4 mm) was excised. The incision was closed and animals were allowed to recover from anesthesia. Laser Doppler tissue imaging showed that obstruction of the left femoral artery decreased blood perfusion by approximately 90% at day 1. After surgery the animals were split into two treatment groups: vehicle, or ixmyelocel-T treated (*n* = 4). After 2 weeks the animals received four intramuscular injections totaling 2 × 10^6^ cells or vehicle in the ischemic limb.

### Laser Doppler assessment of blood flow

Hind-limb perfusion of all animals was assessed prior to surgery using a PeriScan PIM 3 (Perimed AB, Stockholm, Sweden) scanning laser Doppler perfusion imaging system. Ischemia was confirmed in all animals immediately following femoral artery ligation. Blood flow was then measured 14, 42, and 56 days after surgery. After blood flow had been imaged three times, the average blood flow was calculated. The perfusion ratio was defined as blood flow in the ligated limb normalized to baseline. For these studies, normalization to baseline was used since treatment with ixmyelocel-T resulted in increased blood flow in the nonischemic limb, thus not allowing normalization to the nonischemic limb.

### Capillary density

To detect capillary endothelial cells, the frozen sections of the quadriceps muscles (*n* = 4) were stained with CD31 (Santa Cruz, California, CA). Briefly, ischemic limb muscles were harvested at day 56 after treatment and embedded in optimal cutting temperature compound (Tissue-Tek; Sakura, Torrance, California, USA) compound. Frozen sections were stained with CD31 to examine capillary density. Three fields were randomly selected to calculate the number of capillaries in each field. Capillary density was expressed as number of capillaries per myocyte.

### Assessment of marker in plasma

Blood was collected at time of sacrifice. Plasma nitrates and thiobarbituric acid reactive substances (TBARS) were measured in the collected blood plasma using the Cayman Chemical Nitrate Fluorometric Assay (Cayman Chemical, Ann Arbor, MI, USA) and the Cayman Chemical TBARS Assay. Enzyme-linked immunosorbent assay (ELISA) kits were used to determine the concentrations of platelet-derived growth factor (PDGF)-BB and interleukin (IL)-10 (*n* ≥3; R&D Systems, Minneapolis, MN, USA).

### Survival/engraftment of ixmyelocel-T in ischemic hind limb

For detection of transplanted ixmyelocel-T, the cells were labeled with PKH26 (Sigma, St Louis, Mo, USA), a fluorescent cell linker dye, following the manufacturer’s protocol. Nude rats received intramuscular injections of 2 × 10^6^ PKH26-labeled ixmyelocel-T in the ischemic limb. After 8 days the ischemic muscles were excised, embedded in optimal cutting temperature compound, and visualized for PKH26-labeled cells. Frozen tissue sections were also stained with human specific anti-nuclei monoclonal antibody (Chemicon Merck Millipore, Darmstadt, Germany). Primary antibody was incubated for 30 minutes at room temperature prior to incubation with fluorochrome-tagged secondary antibody for 30 minutes. Counterstaining was performed with 4′-6-diamidino-2-phenylindole to visualize nuclei. Fluorescent images (*n* = 4) were visualized using a Nikon Eclipse 80i (Nikon, Melville, NY, USA) equipped with an EXi Aqua Bio-Imaging Microscopy Camera (Q Imaging, Surrey, BC, Canada).

### Flow cytometry

For cell surface staining, erythrocytes were lysed with lysing solution for 10 minutes (Becton Dickinson, San Jose, CA, USA). Fc receptors were blocked with Fc receptor blocking agent (Miltenyi Biotech, Auburn, CA, USA) for 15 minutes at 4°C. Cells were then incubated with surface receptor antibodies for 15 minutes at 4°C, and then washed with phosphate-buffered saline. Cell surface staining was analyzed using the Gallios flow cytometer (Beckman Coulter, Brea, CA, USA). Kaluza software (Beckman Coulter) was used to analyze the acquired data.

### Co-culture

Noncontacting co-culture transwell cell culture systems were developed in order to study the cross-biological activity of HUVECs and ixmyelocel-T. This co-culture system allowed for bidirectional diffusion of soluble factors. The co-cultured cells were prepared as follows. HUVECs were plated on the bottom of cell culture plates (Costar; Sigma Aldrich, St. Louis, MO, USA) and allowed to adhere in EGM-2 (Lonza Inc.). The cells were then washed with serum-free medium and incubated overnight in growth factor-free endothelial cell base media-2 (EBM-2; Lonza Inc.) with 0.05% fetal bovine serum. This served as a control medium throughout the co-culture experiments. Ixmyelocel-T was plated separately in transwell inserts (pore size 0.4 μm; Corning Inc., Corning, NY, USA) in control medium at a ratio of 1:2 (HUVECs:ixmyelocel-T). The transwell inserts containing ixmyelocel-T were then placed into the plates containing HUVECs to initiate the experiments. Co-culture plates were then incubated at 37°C in a humidified air/carbon dioxide (95:5, v/v) atmosphere for 2 or 24 hours. After the incubation period, the co-culture inserts were removed and discarded. The HUVECs were then used in the following experiments.

### Enzyme-linked immunosorbent assay

ELISA kits were used to determine the concentrations of intracellular endothelial nitric oxide synthase (eNOS; R&D Systems). Supernatants from six-well plates containing the HUVECs were collected after the cell culture insert was removed. Intracellular analysis of eNOS was carried out according to the manufacturer’s instructions (*n* ≥11). In brief, HUVECs were washed with phosphate-buffered saline and lysed with R&D cell lysis buffer (R&D Systems). The cell lysates were centrifuged and the supernatants were then assayed following the manufacturer’s instructions.

### Nitric oxide synthase assay and nitrates

Nitric oxide synthase (NOS) was measured using the NOS detection kit (Cell Technology Inc, Mountain View, CA, USA). HUVECs were loaded with diaminofluorescein-2 diacetate, a nonfluorescent cell-permeable reagent that can measure free nitric oxide (NO) and NOS activity in living cells, for 1 hour at 37°C. HUVECs were then co-cultured with ixmyelocel-T or medium (control). Intracellular NOS activity was then measured following the manufacturer’s instructions (*n* ≥9). Nitrates were measured in cell culture supernatants using the Cayman Chemical Nitrate Fluorometric Assay (*n* ≥8).

### Intracellular reactive oxygen species assay

Intracellular reactive oxygen species (ROS) generation was measured using a commercially available intracellular ROS assay (Cell Biolabs Inc., San Diego, CA, USA). Briefly, HUVECs were loaded with the fluorescent probe DCFH-DA for 1 hour at 37°C. HUVECs were then stimulated with 10 ng/ml tumor necrosis factor alpha (TNFα; R&D Systems) for 4 hours. The concentration of TNFα used to induce inflammation and oxidative stress was based upon our preliminary dose-finding study and the previous research of other laboratories [11,12], which demonstrate that 10 ng/ml TNFα significantly increase endothelial oxidative stress and inflammation. The cells were then washed with serum-free medium, and co-cultured with ixmyelocel-T or medium (control) for 2 hours. Intracellular levels of ROS were then measured following the manufacturer’s instructions (*n* ≥6).

### Measurement of intracellular superoxide dismutase activity

Intracellular superoxide dismutase (SOD) was measured using a commercially available SOD Assay (*n* ≥4; Cell Biolabs Inc.). Briefly, HUVECs were stimulated with 10 ng/ml TNFα (R&D Systems) for 4 hours. The cells were then washed with serum-free medium, and co-cultured with ixmyelocel-T or medium (control) for 2 hours. Intracellular levels of SOD were then measured following the manufacturer’s instructions.

### Immunofluorescence

Immunofluorescence of eNOS was evaluated in HUVECs co-cultured with ixmyelocel-T or medium (control) (*n* ≥3). Briefly, HUVECs were grown on coverslips in six-well culture dishes. Ixmyelocel-T or medium (control) were then placed in co-culture inserts and co-cultured with the HUVECs for 2 hours. The co-cultures were then removed, and the HUVECs were fixed with 10% formalin for 10 minutes. Each cover slip was stained with eNOS antibody (Santa Cruz Biotechnology Inc., Santa Cruz, CA, USA) for 2 hours at room temperature prior to incubation with fluorochrome-tagged secondary antibody for 30 minutes. Counterstaining was performed with 4′-6-diamidino-2-phenylindole to visualize nuclei. Fluorescent images were visualized using a Nikon Eclipse 80i equipped with an EXi Aqua Bio-Imaging Microscopy Camera (Q Imaging).

### Assessment of apoptosis

Apoptosis was measured in HUVECs that were plated in 96-well plates, starved overnight in control medium, followed by induction of apoptosis with 100 ng/ml TNFα (R&D Systems) for 6 hours (*n* = 6). The concentration of TNFα used to induce apoptosis was based upon our preliminary dose-finding study and previous findings [13]. Viability and induction of apoptosis were examined by measuring caspase-3 and caspase-7 activity using the Apo-Tox assay (Promega, Wallisellen, Switzerland). Both fluorescence and luminescence units were measured using a SpectraMax plate reader (Molecular Devices, Sunnyvale, CA, USA) and values were expressed relative to those obtained from the HUVEC control group.

### Real-time PCR

For real-time PCR, total RNA was extracted with an RNeasy Mini Kit (Qiagen, Valencia, CA, USA) and 1 μg RNA was reverse transcribed using a high-capacity cDNA reverse transcription kit (Applied Biosciences, Carlsbad, CA, USA). Relative levels of target gene expression were measured on the 7500 Real-Time PCR system (Applied Biosystems). The FAM-based Taqman Gene Expression Assay Mix (Applied Biosystems) specific for each gene of interest and Taqman Universal Master Mix (Applied Biosystems) were used. Relative quantification PCR analysis was performed using the ABI 7500 Software (Applied Biosystems). The relative amount of cDNA was calculated by normalization to glyceraldehyde 3-phosphate dehydrogenase.

### Proliferation assay

HUVECs were seeded at a density of 5 × 10^3^ cells per well in 96 well transwell insert plates (Corning Inc.) and co-cultured with varying concentrations of ixmyelocel-T plated in inserts for 72 hours (*n* ≥4). Proliferation was measured by incubation with bromodeoxyuridine at a final concentration of 10 mM. Bromodeoxyuridine incorporation was measured by a commercially available ELISA (Cell Signaling Technology, Danvers, MA, USA).

### *In vitro* scratch assay

HUVECs were seeded in 24-well plates and allowed to form confluent monolayers. Cells were then cultured in serum starvation media (EBM-2 + 0.05% fetal bovine serum) overnight. Monolayers were scratched using a sterile 200 μl pipette tip. Cells were washed with phosphate-buffered saline, and fresh serum starvation media (EBM-2 + 0.05% fetal bovine serum) was replaced. Images were taken for the starting point of each scratch using a Nikon Eclipse TE2000-S Microscope (Nikon, Tokyo, Japan) equipped with a Spot Xplorer Leica digital camera (SPOT, Sterling Heights, MI, USA). Ixmyelocel-T was plated in transwell cell culture plates (pore size 0.4 μm; Corning Inc.) in serum-free HUVEC media. The plate was then placed in an incubator for 16 hours, after which scratches were imaged. Changes in wound width were calculated using ImageJ software (National Institutes of Health, Besthesda, Maryland, USA). Assays were performed in triplicate (*n* ≥4).

### Matrigel assays

HUVECs were seeded at a density of 5 × 10^4^ cells per well in 24-well Matrigel-coated (BD Bioscience) plates. Ixmyelocel-T was plated onto Transwell inserts (24-well clusters, 0.4 μm pore size; Corning Inc.) at varying densities. Tube branch formation and length was measured after 8 hours of co-culture in three random microscopic fields taken at 10× magnification (*n* ≥4). Tube branch formation and length were quantified with the ImageJ analysis software.

### Cytokine arrays

To identify angiogenic factors secreted by HUVECs and by HUVECs co-cultured with ixmyelocel-T, the protein levels of specific factors in cell-conditioned medium were measured (*n* ≥3). Cytokine arrays were performed according to manufacturer’s protocols (RayBiotech Inc., Norcross, Georgia, USA) in conditioned medium. Briefly, HUVECs were stimulated with 10 ng/ml TNFα (R&D Systems) for 4 hours. The cells were then washed with serum-free medium, and co-cultured with ixmyelocel-T or medium (control) for 24 hours, after which the supernatants were collected for analysis.

### Statistical analysis

A paired *t* test was performed to compare results. *P* <0.05 was considered statistically significant. Data are reported as mean ± standard error of the mean.

## Results

### Ixmyelocel-T contains expanded populations of regenerative cells

**Figure 1:**
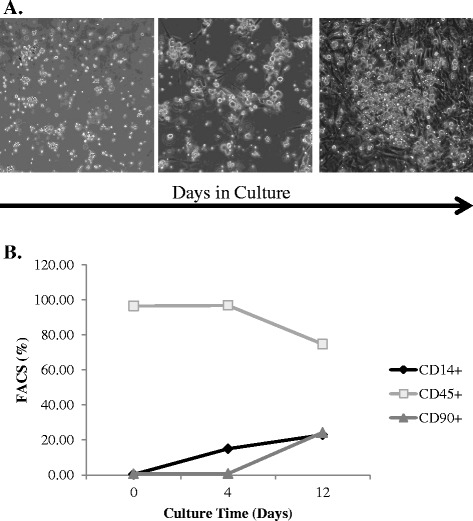
**Ixmyelocel-T is a multicellular therapy containing expanded populations of CD90** ^**+**^ **mesenchymal stem cells and CD14** ^**+**^ **M2-like macrophages. (A)** Phase imaging depicts the expansion of cells during the culture process. **(B)** Kinetics of ixmyelocel-T culture. Magnification: 20×. FACS, fluorescence-activated cell sorting.

Ixmyelocel-T is composed of a mixture of cells, including myeloid cells, lymphoid cells, and MSCs/stromal cells [7]. As reported previously, the two main cell types expanded from BMMNCs in Aastrom’s manufacturing process are CD90^+^ MSCs and CD14^+^ macrophages [7]. Figure 1 displays microscopy images of ixmyelocel-T taken throughout the culture process to highlight the expansion that occurs during culture (Figure 1A), and flow cytometry analysis highlights the two regenerative populations that are expanded in this culture process (Figure 1B).

### Ixmyelocel-T secretes multiple angiogenic cytokines and growth factors when co-cultured with endothelial cells

Since it has been reported previously that the benefits of cell therapies, such as angiogenesis, are mainly due to paracrine effects [4], a cytokine array was utilized to determine what proangiogenic factors ixmyelocel-T secretes during co-culture with endothelial cells. Secretion of several proangiogenic and proliferative factors were quantified using a cytokine array (Table 1). Specifically, the cytokines IL-3, IL-6, and IL-8, the chemokines CXCL5, CCL1, and CCL7, macrophage migration inhibitory factor, and growth factors PDGF-BB, epidermal growth factor, hepatocyte growth factor, placental growth factor, angiogenin, and osteoprotegerin were elevated in the supernatants from the HUVECs co-cultured with ixmyelocel-T (Table 1). These results suggest that ixmyelocel-T has the potential to promote angiogenesis and vascular repair through the secretion of a variety of proangiogenic factors.

**Table 1:** **Cytokine secretion**

	**HUVECs**	**IXT**	**HUVECs + IXT**
Interleukin-3	181 ± 15	240 ± 20*	229 ± 24
Interleukin-6	9,610 ± 808	9,201 ± 7,368	59,567 ± 3,801***^,‡^
Interleukin-8	16,525 ± 1,697	36,998 ± 5,957**	35,128 ± 9,227
CXCL5	205 ± 34	7,893 ± 4,630	9,385 ± 1,710**
CCL1	83 ± 3	28 ± 8***	204 ± 39*^,†^
CCL7	67 ± 11	10,563 ± 1,748**	13,901 ± 3821**
MIF	3,765 ± 761	5,234 ± 1,556	5,364 ± 1,114
VEGF	15 ± 4	428 ± 275	95 ± 57
PDGF-BB	225 ± 29	34 ± 2***	1,065 ± 345*^,†^
EGF	171 ± 51	17 ± 5*	256 ± 74^†^
HGF	51 ± 8	5,752 ± 2,324*	271 ± 40**^,†^
PlFG	1,218 ± 210	1,123 ± 476^†^	2,648 ± 477*
Angiogenin	17,13 ± 237	33,102 ± 7,300**	8,722 ± 3,384*^,†^
Osteoprotegerin	434 ± 5	8,041 ± 5,992	2,049 ± 1,468

Cytokines were quantified in supernatants from tumor necrosis factor alpha pretreated HUVECs, HUVECS co-cultured with ixmyelocel-T, and ixmyelocel-T (*n* ≥3). Values are presented as mean ± standard error of the mean relative to control. EGF, epidermal growth factor; HGF, hepatocyte growth factor; HUVEC, human umbilical vein endothelial cell; IXT, ixmyelocel-T; MIF, macrophage migration inhibitory factor; PDGF, platelet-derived growth factor; PlGF, placental growth factor; VEGF, vascular endothelial growth factor. *P <0.05, **P <0.01, ***P <0.001 versus HUVEC. ^†^ *P* <0.05, ^‡^ *P* <0.01 versus IXT.

### Ixmyelocel-T promotes the angiogenic capacity of endothelial cells *in vitro*

**Figure 2:**
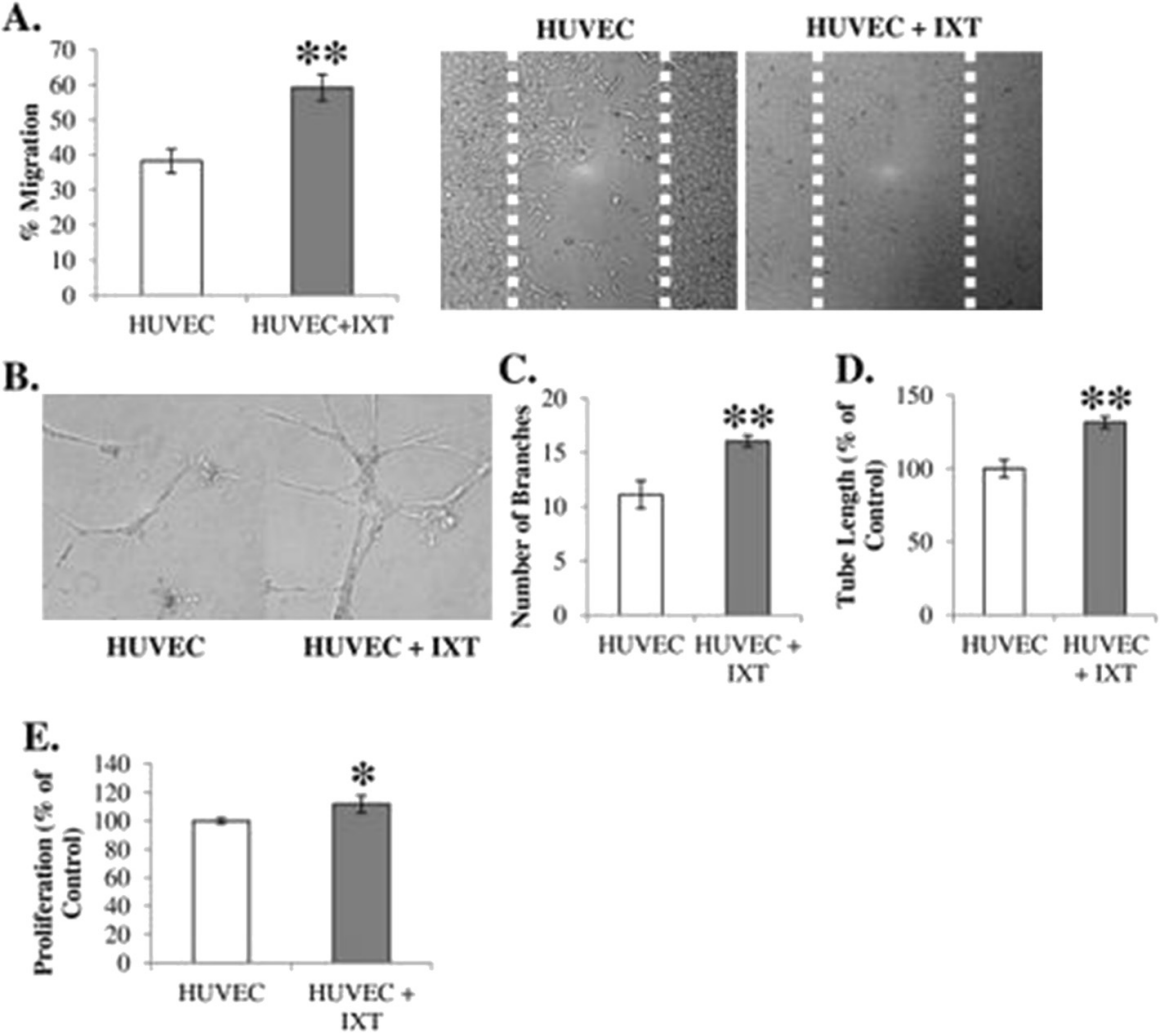
**Ixmyelocel-T promotes the angiogenic ability of endothelial cells** ***in vitro*** **. (A)** Ixmyelocel-T promotes migration of human umbilical vein endothelial cells (HUVECs) measured with the *in vitro* scratch assay (magnification 10×). **(B** **, C,** **D)** Ixmyelocel-T co-culture with HUVECs on Matrigel induced increased HUVEC branch formation and length (magnification 20×). **(E)** Ixmyelocel-T promotes the proliferation of HUVECs (*n* ≥4). Values presented as mean ± standard error of the mean relative to control. *P <0.05, **P <0.001 versus HUVECs. IXT, ixmyelocel-T.

To determine whether ixmyelocel-T promoted the angiogenic activity of endothelial cells, ixmyelocel-T was co-cultured with HUVECs using noncontacting co-culture inserts. To determine whether ixmyelocel-T promotes endothelial cell migration, an *in vitro* scratch assay was employed. HUVEC co-cultures with ixmyelocel-T exhibited significantly increased cell migration (59 ± 3.7 vs. 38 ± 3.4% migration, *P* <0.001 compared with HUVECs; Figure 2A). The effect of ixmyelocel-T on the induction of capillary-like structures (angiogenesis and neovascularization) *in vitro* was examined using a well-established model – the Matrigel *in vitro* angiogenesis assay. HUEVCS were grown on Matrigel-coated plates in serum-free conditions. Co-culture with ixmyelocel-T increased branch formation (16 ± 0.5 vs. 11 ± 1.3 branch number, *P* <0.01 compared with HUVECs; Figure 2B-C) and tube length (131 ± 4 vs. 100 ± 6%, *P* <0.001 compared with HUVECs; Figure 2D). To determine whether ixmyelocel-T promotes endothelial proliferation, ixmyelocel-T was co-cultured with HUVECs and analyzed using a bromodeoxyuridine proliferation assay. Co-culture of HUVECs with ixmyelocel-T resulted in significantly increased endothelial proliferation (112 ± 6 vs. 100 ± 2% of HUVECs, *P* <0.05 compared with HUVECs; Figure 2E).

### Co-culture of ixmyelocel-T with endothelial cells results in increased eNOS expression and nitric oxide production

**Figure 3:**
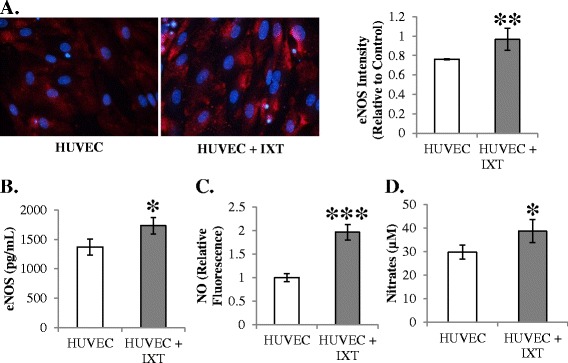
**Ixmyelocel-T co-culture results in increased endothelial nitric oxide synthase and nitric oxide production in endothelial cells. (A)** Immunofluorescence of endothelial nitric oxide synthase (eNOS) in co-cultured human umbilical vein endothelial cells (HUVECs) (*n* ≥3). **(B)** eNOS protein (*n* ≥11). **(C)** Nitric oxide (NO) generation (*n* ≥9). **(D)** Nitrates measured in co-culture supernatants (*n* ≥8). Values presented as mean ± standard error of the mean relative to control. *P <0.05, **P <0.01, ***P <0.001 versus HUVECs. Magnification: 20×. IXT, ixmyelocel-T.

NO regulates blood flow recovery after ischemia and exerts multiple signaling events resulting in endothelial proliferation, migration, and survival [14]. HUVECs were co-cultured with ixmyelocel-T and eNOS expression was examined. Immunofluorescence of eNOS was significantly greater in HUVECs co-cultured with ixmyelocel-T compared with HUVECs alone (1.3 ± 0.11 vs. 1.0 ± 0.01 relative intensity, *P* <0.01; Figure 3A). There were also statistically significant higher intracellular levels of eNOS in HUVECs co-cultured with ixmyelocel-T compared with HUVECs alone (1,730 ± 141 vs. 1,371 ± 135 pg/ml, *P* <0.05; Figure 3B) measured by ELISA. NO production was assessed with the intracellular probe diaminofluorescein-2 diacetate. HUVECs co-cultured with ixmyelocel-T displayed a statistically significant increase in NO production compared with HUVECs alone (1.97 ± 0.17 vs. 1 ± 0.09 relative fluorescence, *P* <0.001; Figure 3C). Nitrates were also measured in the supernatants of the co-cultured cells as a marker of NO production. HUVECs co-cultured with ixmyelocel-T had statistically significant increased levels of nitrates in their supernatants compared with HUVECs (39 ± 5 vs. 30 ± 3 μM, *P* <0.05; Figure 3D). These data suggest that ixmyelocel-T promotes NO production in endothelial cells.

### Ixmyelocel-T attenuates apoptosis and inflammation when co-cultured with TNFα-stimulated endothelial cells

**Figure 4:**
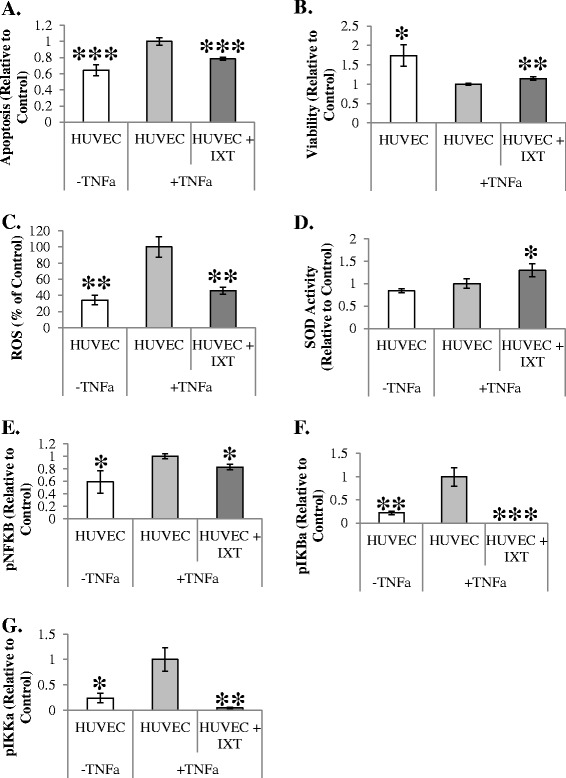
**Co-culture with ixmyelocel-T decreases apoptosis, oxidative stress, and inflammation in TNFα** **pretreated endothelial cells. (A)** Apoptosis (*n* = 6). **(B)** Viability was decreased with ixmyelocel-T co-culture (*n* = 6). **(C)** Ixmyelocel-T co-culture decreased reactive oxygen species (ROS) generation (*n* ≥6). **(D)** Ixmyelocel-T co-culture increased antioxidant superoxide dismutase (SOD) in human umbilical vein endothelial cells (HUVECs) after tumor necrosis factor alpha (TNFα) stimulation (*n* ≥4). Protein expression of **(E)** pNFκB, **(F)** pIKBa, and **(G)** pIKKa were all significantly decreased in HUVECs co-cultured with ixmyelocel-T after TNFα stimulation (*n* = 3). Values presented as mean ± standard error of the mean relative to control. *P <0.05, **P <0.01, ***P <0.001 versus HUVECs + TNFα. IXT, ixmyelocel-T.

Ixmyelocel-T was characterized previously with anti-inflammatory properties [7,15], and thus the effects of ixmyelocel-T on proinflammatory stimulated endothelial cells was examined. Apoptosis of endothelial cells disrupts the integrity of the endothelial monolayer, contributing to the initiation of proinflammatory events [16]. To induce vascular injury, HUVECs were pretreated with TNFα. This TNFα stimulation increased endothelial cell apoptosis (0.64 ± 0.07 vs. 1 ± 0.05 relative to HUVECs, *P* <0.001 compared with HUVECs + TNFα; Figure 4A) and decreased cell viability (1.74 ± 0.27 vs. 1 ± 0.03 relative to HUVECs, *P* <0.05 compared with HUVECs + TNFα; Figure 4B). Co-culture with ixmyelocel-T resulted in a statistically significant increase in HUVEC viability (1.14 ± 0.04 vs. 1 ± 0.03 relative to HUVECs, *P* <0.01 compared with HUVECs + TNFα; Figure 4B) and decrease in HUVEC apoptosis (0.78 ± 0.02 vs. 1 ± 0.05 relative to HUVECs, *P* <0.001 compared with HUVECs + TNFα; Figure 4A). ROS generation was also examined in HUVECs stimulated with TNFα by measuring the intensity of DCFH-DA fluorescence. There was a statistically significant increase in ROS concentration in HUVECs treated with TNFα (34 ± 6% vs. 100 ± 26% of HUVECs, *P* <0.01 compared with HUVECs + TNFα; Figure 4C). Co-culture with ixmyelocel-T attenuated the TNFα-induced increase in ROS concentration in HUVECs (46 ± 4% vs. 100 ± 26% of HUVECs, *P* <0.01 compared with HUVECs + TNFα; Figure 4C). Ixmyelocel-T co-culture also resulted in a statistically significant increase in the activity of the antioxidant enzyme SOD in TNFα-stimulated HUVECs (1.3 ± 0.1% vs. 1 ± 0.1% of HUVECs, *P* <0.05 compared with HUVECs + TNFα; Figure 4D). Altogether these data suggest that ixmyelocel-T has the ability to decrease oxidative stress in endothelial cells.

Nuclear factor (NF)-κB is a transcription factor that regulates the expression of proinflammatory and anti-apoptotic genes [17]. NF-κB activation was therefore determined using an activation ELISA in HUVECs pretreated with TNFα followed by co-culture with ixmyelocel-T in order to determine whether ixmyelocel-T secreted factors act on this pathway. Co-culture with ixmyelocel-T attenuated the TNFα-induced increase of pNF-κB (0.83 ± 0.04 vs. 1 ± 0.04 relative to control, *P* <0.05 compared with HUVECs + TNFα; Figure 4D), pIKBα (0.83 ± 0.0 vs. 1 ± 0.2 relative to control, *P* <0.001 compared with HUVECs + TNFα; Figure 4E), and pIKKα (0.04 ± 0.02 vs. 1 ± 0.23 relative to control, *P* <0.01 compared with HUVECs + TNFα; Figure 4F) in HUVECs. Together these data suggest that ixmyelocel-T has the ability to dampen the inflammatory response in endothelial cells in a paracrine manner.

### Ixmyelocel-T promotes blood flow recovery in the hind-limb model

**Figure 5:**
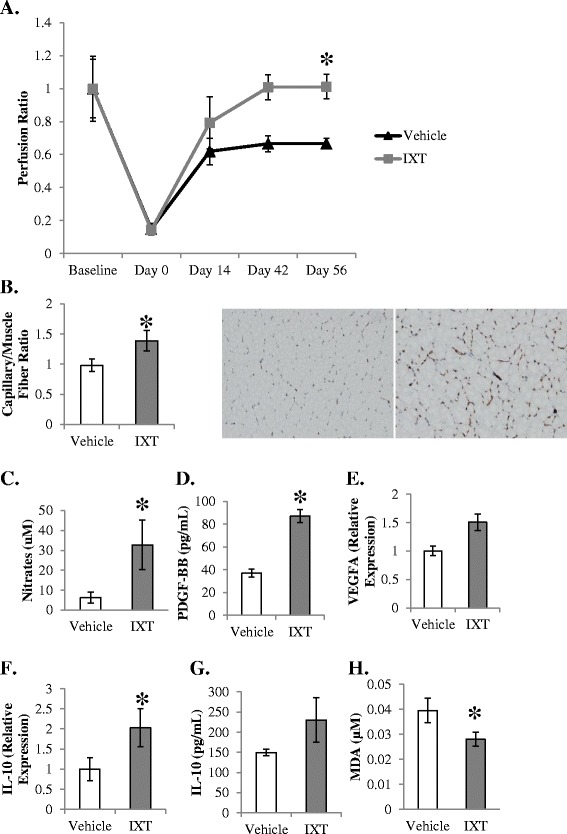
**Ixmyelocel-T promotes blood flow recovery in the rat hind-limb model. (A)** Treatment with ixmyelocel-T significantly increased blood flow recovery measured by laser Doppler (*n* = 4). **(B)** Treatment with ixmyelocel-T significantly increased capillary density in the ischemic limb (10×) (*n* = 4). (**C,** **D**) Treatment with ixmyelocel-T significantly increased plasma nitrates (*n* = 4) and platelet-derived growth factor (PDGF; *n* = 3). **(E)** Treatment with ixmyelocel-T increased gene expression of vascular endothelial growth factor (VEGF) in the ischemic limb (*n* = 4). **(F)** Gene expression of the anti-inflammatory cytokine interleukin (IL)-10 is significantly increased in the ischemic limb of the ixmyelocel-T-treated group (*n* = 4). **(G)** Circulating levels of IL-10 trended upwards in the ixmyelocel-T-treated group (*n* = 4). **(H)** Plasma levels of thiobarbituric acid reactive substances were significantly decreased in the ixmyelocel-T-treated group (*n* = 4). Values presented as mean ± standard error of the mean. *P <0.05 versus vehicle. Magnification: 10×. IXT, ixmyelocel-T; MDA, malondialdehyde.

Although rats in the control group display a compensatory recovery of blood flow recovery, treatment with ixmyelocel-T promoted remarkable restoration of blood flow, as the perfusion ratio in the ixmyelocel-T group was significantly greater than in the control group after 8 weeks (1.01 ± 0.07 vs. 0.67 ± 0.03 perfusion ratio, *P* <0.05 compared with vehicle; Figure 5A). Consistently, immunohistochemistry revealed that the CD31^+^ capillary to muscle fiber ratio was significantly higher in the rats receiving ixmyelocel-T (1.39 ± 0.2 vs. 0.98 ± 0.1 capillary/muscle fiber ratio, *P* <0.05 compared with vehicle; Figure 5B). Treatment with ixmyelocel-T also resulted in increased plasma nitrates (32.75 ± 12.44 vs. 6.23 ± 2.84 μM, *P* <0.05 compared with vehicle; Figure 5C), indicating that treatment with ixmyelocel-T resulted in increased NO bioavailability Plasma levels of the angiogenic growth factor PDGF-BB were significantly elevated in the ixmyelocel-T-treated group (87 ± 5.9 vs. 37 ± 3.4 pg/ml, *P* <0.001 compared with vehicle; Figure 5D). Gene expression of *VEGFA* was also elevated in the ischemic limb of the ixmyelocel-T-treated group (1.5 ± 0.14 vs. 1.0 ± 0.08 relative to control, *P* <0.01 compared with vehicle; Figure 5E). Together these data suggest that ixmyelocel-T promotes angiogenesis in ischemic tissue. Since ixmyelocel-T has been reported previously to have anti-inflammatory properties, markers of inflammation were examined in the ischemic limbs. Gene expression of the anti-inflammatory marker *IL-10* was increased in the ischemic limb (2.0 ± 0.47 vs. 1.0 ± 0.29 relative to control, *P* <0.05 compared with vehicle; Figure 5F) and plasma levels of IL-10 trended upwards (230 ± 55 vs. 149 ± 7.9 pg/ml; Figure 5G) in the ixmyelocel-T-treated group. Plasma levels of the oxidative stress marker TBARS were significantly decreased in the ixmyelocel-T-treated group (0.03 ± 0.003 vs. 0.04 ± 0.005 μM, *P* <0.05 compared with vehicle; Figure 5H).

### Survival of transplanted ixmyelocel-T in ischemic limb muscles

**Figure 6:**
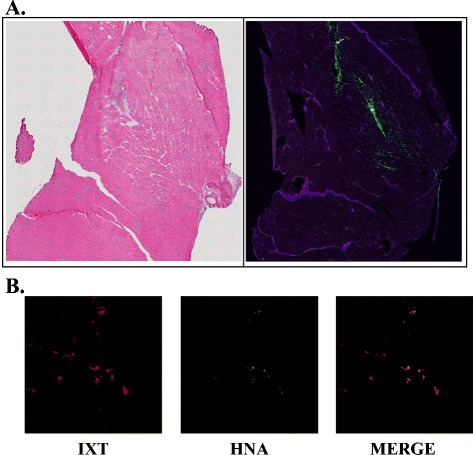
**Analysis of PKH26-labeled ixmyelocel-T in the hind-limb ischemia model. (A)** High magnification of labeled ixmyelocel-T (green) throughout the ischemic limb 8 days after initiation of hind-limb ischemia. Sections were immunostained for type I collagen (purple) and counterstained with 4′-6-diamidino-2-phenylindole (Blue). **(B)** Analysis of ixmyelocel-T (red) co-localization with human nuclear antigen (green) in the ischemic rodent limb. Magnification: 400×. HNA, human nuclear antigen; IXT, ixmyelocel-T.

Engraftment and the survival potential of ixmyelocel-T were examined in the ischemic hind-limb model. Ixmyelocel-T was labeled with the stable-cell, fluorescent tracking dye PKH-26. PKH-positive cells were detected in the ischemic limb 8 days after transplantation (Figure 6A). PKH labeling can be often be transferred to host cells when it is taken up by phagocytosis of labeled apoptotic cells. To confirm that the PKH-positive cells were of human origin, immuofluorescent staining of human nuclear antigen, which specifically labels human cells, was performed (Figure 6B). Dual-positive PKH-labeled and HNA-positive cells were detected in the ischemic limbs. These data suggest that ixmyelocel-T persists in the ischemic tissue and promotes angiogenesis and regeneration in ischemia.

## Discussion

This study demonstrates that bone marrow-derived expanded ixmyelocel-T has angiogenic potential which may be useful for recovering tissues from ischemia. Ixmyelocel-T was demonstrated to interact with endothelial cells in a paracrine manner through secretion of angiogenic growth factors that promote therapeutic angiogenesis and endothelial repair. This study highlighted the effects ixmyelocel-T exerts on endothelial cells. Specifically, HUVECs stimulated by angiogenic factors secreted by ixmyelocel-T exhibited enhanced migration, proliferation, and tube formation. Co-culture of HUVECs with ixmyelocel-T also resulted in significantly increased NO bioavailability, decreased markers of ROS and inflammation, and reduced apoptosis. Most importantly, ixmyelocel-T promoted significant blood flow recovery which was maintained for up to 8 weeks after treatment, and induced capillary formation in a rat hind-limb ischemia model. All together these data suggest that ixmyelocel-T could be useful for the promoting of angiogenesis and tissue repair in ischemic CV diseases.

Soluble factors play a pivotal role in influencing cells within the local microenvironment by promoting cell survival and activating tissue repair [5]. Using an antibody array, we identified a panel of proangiogenic cytokine and growth factors differentially secreted in co-cultures of HUVECS and ixmyelocel-T. Hepatocyte growth factor, a growth factor that promotes endothelial regeneration [18], was significantly upregulated in the supernatants of HUVECs co-cultured with ixmyelocel-T. IL-6, which protects endothelial cells from oxidative stress-induced apoptosis, was significantly increased in the supernatants of HUVECs co-cultured with ixmyelocel-T [19]. Ixmyelocel-T secreted elevated levels of IL-8, a cytokine secreted by macrophages that promote angiogenesis [20]. Additionally, IL-6 and IL-8 are reported to increase endothelial progenitor cell homing to areas of ischemia [21]. The chemokine CXCL5, which promotes angiogenesis in ischemic tissues through the recruitment of endothelial cells [22], was found significantly elevated in the supernatants of HUVECs co-cultured with ixmyelocel-T. CCL7, a chemokine suggested to improve tissue repair in ischemia by acting as a homing factor for circulating angiogenic cells [23], was also elevated in the supernatant of HUVECs co-cultured with ixmyelocel-T. Macrophage migration inhibitory factor, which promotes angiogenesis through endothelial cell activation and endothelial progenitor cell recruitment [24], was elevated in ixmyelocel-T supernatants. CL1, which stimulates endothelial progenitor cell tube formation [25], was significantly increased in the supernatants of HUVECs co-cultured with ixmyelocel-T. Placental growth factor, an angiogenic factor that stabilizes blood vessels and enhances vascular endothelial growth factor angiogenic activity [26], was significantly upregulated in the supernatants of HUVECs co-cultured with ixmyelocel-T. Angiogenin, an angiogenic factor that triggers eNOS activity resulting in increased NO synthesis [27], was significantly increased in the supernatants of HUVECs co-cultured with ixmyelocel-T. Osteoprotegerin, which enhances the angiogenic effects of ECPs and positively regulates the formation of new vessels [28], was elevated in ixmyelocel-T supernatants. PDGF-BB, which increases endothelial proliferation and migration [29], was significantly upregulated in the supernatants of HUVECs co-cultured with ixmyelocel-T. PDGF-BB was also found to be significantly increased in the blood plasma of the ixmyelocel-T-treated group. Multiple proangiogenic cytokines/growth factors released from the multiple cell populations that make up ixmyelocel-T may synergistically augment neoangiogenesis.

Therapeutic angiogenesis is essential for tissue recovery after injury, and is dependent on endothelial cells [30]. Enhancing endothelial proliferation and angiogenesis may provide a beneficial approach for the ischemic complications of atherosclerotic diseases. To validate the angiogenic activity present in the ixmyelocel-T secretome, various functional assays were utilized. Co-culture of ixmyelocel-T with HUVECs promoted endothelial cell migration, tube formation, and proliferation in *in vitro* through a paracrine manner. eNOS-derived NO is an important mediator of ischemic angiogenesis [14]. Co-culture of HUVECs with ixmyelocel-T increased NO bioavailability by enhancing both eNOS expression measured by immunostaining and ELISA and activation measured with the intracellular NO probe and nitrate formation in the co-culture supernatants. Ixmyelocel-T treatment also significantly increased plasma nitrates in the hind-limb ischemia model. The induction of NO production by ixmyelocel-T has the potential to promote vascular homeostasis, promoting cell growth and vascular protection from injury [31]. Altogether, these results suggest that ixmyelocel-T promotes angiogenesis through promoting endothelial cell proliferation, migration, tube formation, and NO production.

Ischemic CV diseases are the consequence of endothelial damage, and a major case of this damage is oxidative stress and inflammation [30,32]. Oxidative stress has been reported to impair endothelial growth and angiogenesis, and when the ability of the endothelium to handle ROS is exceeded, the resulting oxidative stress leads to apoptosis and subsequent cell death [16,33]. Rats treated in this study with ixmyelocel-T exhibited decreased plasma TBARS, a marker of oxidative stress, and increased expression of the anti-inflammatory cytokine IL-10. Additionally, HUVECs pretreated with TNFα displayed significant increases in intracellular ROS that was significantly decreased in HUVECs co-cultured with ixmyelocel-T. Intracellular antioxidant enzymes maintain the cellular redox homeostasis by preventing excess ROS formation [34,35]. Intracellular levels of ROS are regulated by the balance between ROS-generating enzymes and antioxidant enzymes, such as SOD [35]. SOD plays a pivotal role in preventing cellular damage caused by ROS through reducing superoxide radicals [34]. When co-cultured with HUVECs, ixmyelocel-T was able to increase the activity of SOD, increase cell viability, and decrease apoptosis. Previous studies have demonstrated anti-inflammatory activities of ixmyelocel-T [7,8]. NF-κB, a transcription factor that regulates the expression of proinflammatory and anti-apoptotic markers, plays a significant role in driving the inflammatory response [17,36]. NF-κB is regulated by the association with inhibitory IκB molecules. It can become activated by several different signals including TNFα, which leads to the release of NF-κB from its complex with the IκB molecules [36]. In this study, TNFα treatment resulted in a significant increase in NF-κB activation in HUVECs through the activation of NF-κB, IκBa, and Iκκa. Treatment with ixmyelocel-T significantly reduced NF-κB, IκBa, and Iκκa activation in TNFα-treated HUVECs. The inhibitory effect of ixmyelocel-T on the NF-κB pathway might explain the reduction in ROS formation and the decrease in apoptosis in HUVECs pretreated with TNFα. As a result of these findings, treatment with ixmyelocel-T may prevent endothelial cell apoptosis in a paracrine manner, perhaps due to decreased intracellular ROS, increased antioxidant activity, and decreased NF-κB activity. These characteristics associated with ixmyelocel-T may promote therapeutic effects in the treatment of ischemic CV diseases, where cell survival depends on the ability of cells to overcome apoptotic triggers, especially in ischemic tissue.

The aim of the present study was not to access the benefit of a single factor secreted by the multicellular cell therapy ixmyelocel-T, but rather to examine whether the pleiotropic activity of this mixed population of cells can enhance vessel repair and new vessel growth in ischemic tissue. Tissue regeneration is a complex process involving an interplay between macrophages, stem cells, and stromal cells, and it is hypothesized that a mixture of regenerative cells, rather than just a single cell type, might be more advantageous [7,37]. Ixmyelocel-T consists of a mixture of cells expanded from BMMNCs, specifically an expanded population of CD90^+^ stromal cells and CD14^+^ macrophages that have been characterized with a M2-like phenotype [7]. It is thought that the mixture of cells found in ixmyelocel-T might be more advantageous in long-term tissue regeneration and repair [7,37]. Specifically, it has been reported that MSCs secrete factors that regulate angiogenesis, and it has been demonstrated that MSCs promote angiogenesis after hind-limb ischemia through secretion of growth factors [38]. The contribution of myeloid cells in angiogenesis has also been established previously [39,40]. Monocytes/macrophages promote angiogenesis through the secretion of angiogenic factors and ECM remodeling [39]. The novelty and advantage of using ixmyelocel-T are due to its manufacturing process, which relies on a small volume of bone marrow aspirate, the unique combination of cell populations including expanded MSCS and M2-like macrophages, the secretion of a distinct combination of angiogenic and regenerative factors, the ability to remain anti-inflammatory in the face of inflammatory challenge, and the substantial angiogenic capacity associated with the therapy. These beneficial effects position ixmyelocel-T as an optimal cell therapy for treating severe ischemic CV diseases where treatment options are limited.

## Conclusions

The present study demonstrates that ixmyelocel-T, a bone marrow-derived expanded autologous multicellular therapy, promotes blood flow recovery and angiogenesis *in vivo* and interacts with human endothelial cells *in vitro* in a paracrine manner by enhancing migration, proliferation, and branch formation. Taken together, these data support a role for indirect angiogenesis and endothelial cell protection from ischemic injury as at least a part of the putative mechanism of action in ischemic CV patients.

In conclusion, although the use of therapeutic angiogenesis with BMMNCs in CLI patients has been established, ixmyelocel-T therapy may provide a new aspect of therapeutic angiogenesis in this patient population where expanded populations of regenerative cells might be required.

## Abbreviations

BMMNC: bone marrow mononuclear cell
CLI: critical limb ischemia
CV: cardiovascular
ELISA: enzyme-linked immunosorbent assay
eNOS: endothelial nitric oxide synthase
HUVEC: human umbilical vein endothelial cell
IL: interleukin
MSC: mesenchymal stromal cell
NF: nuclear factor
NO: nitric oxide
NOS: nitric oxide synthase
PDGF: platelet-derived growth factor
ROS: reactive oxygen species
SOD: superoxide dismutase
TBARS: thiobarbituric acid reactive substances
TNFα: tumor necrosis factor alpha
